# *catena*-Poly[[[aqua­bis­(4,4′-bipyridine-κ*N*)zinc]-μ-l-tyrosinato-κ^3^ *N*,*O* ^1^:*O* ^1′^] nitrate dihydrate]

**DOI:** 10.1107/S1600536811021088

**Published:** 2011-06-11

**Authors:** Shu-Qiang Li, Ning-Hai Hu

**Affiliations:** a Orthopaedic Department, First Hospital, Jilin University, Changchun 130021, People’s Republic of China; b Changchun Institute of Applied Chemistry, Chinese Academy of Sciences, Changchun 130022, People’s Republic of China

## Abstract

In the title compound, {[Zn(C_9_H_10_NO_3_)(C_10_H_8_N_2_)_2_(H_2_O)]NO_3_·2H_2_O}_*n*_, the Zn^II^ atom is six-coordinated in a distorted octa­hedral geometry by two carboxyl­ate O atoms and one amino N atom from two l-tyrosinate ligands, two N atoms from two 4,4′-bipyridine ligands, and one water mol­ecule. Adjacent Zn atoms are bridged by the bidentate carboxyl­ate groups into a cationic chain extending along [010]. N—H⋯N, O—H⋯N and O—H⋯O hydrogen bonds link the cationic chains, nitrate anions and uncoordinated water mol­ecules into a supra­molecular network. π–π inter­actions between the pyridine rings and between the pyridine and benzene rings [centroid–centroid distances = 3.615 (4) and 3.636 (4) Å] are present.

## Related literature

For general background to the structures and properties of chiral coordination polymers, see: Dai *et al.* (2005 ▶); Kesanli & Lin (2003 ▶); Vaidhyanathan *et al.* (2006 ▶); Zaworotko (2001 ▶). For related structures, see: Lou & Hong (2008 ▶); Lou *et al.* (2005 ▶, 2007 ▶); Zhang & Hu (2009 ▶).
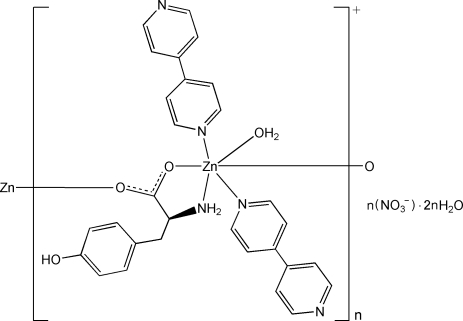


## Experimental

### 

#### Crystal data

[Zn(C_9_H_10_NO_3_)(C_10_H_8_N_2_)_2_(H_2_O)]NO_3_·2H_2_O
*M* *_r_* = 673.98
Monoclinic, 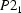

*a* = 12.737 (3) Å
*b* = 10.351 (2) Å
*c* = 12.921 (3) Å
β = 117.897 (5)°
*V* = 1505.5 (6) Å^3^
*Z* = 2
Mo *K*α radiation
μ = 0.88 mm^−1^
*T* = 293 K
0.22 × 0.09 × 0.02 mm

#### Data collection

Bruker APEX CCD diffractometer
Absorption correction: multi-scan (*SADABS*; Sheldrick, 1996 ▶) *T*_min_ = 0.830, *T*_max_ = 0.983
7908 measured reflections
5199 independent reflections
3621 reflections with *I* > 2σ(*I*)
*R*_int_ = 0.048

#### Refinement

*R*[*F* ^2^ > 2σ(*F* ^2^)] = 0.056
*wR*(*F* ^2^) = 0.091
*S* = 0.97
5199 reflections
406 parameters
1 restraint
H-atom parameters constrained
Δρ_max_ = 0.47 e Å^−3^
Δρ_min_ = −0.42 e Å^−3^
Absolute structure: Flack (1983 ▶), 2352 Friedel pairs
Flack parameter: 0.045 (14)

### 

Data collection: *SMART* (Bruker, 2007 ▶); cell refinement: *SAINT* (Bruker, 2007 ▶); data reduction: *SAINT*; program(s) used to solve structure: *SHELXS97* (Sheldrick, 2008 ▶); program(s) used to refine structure: *SHELXL97* (Sheldrick, 2008 ▶); molecular graphics: *SHELXTL* (Sheldrick, 2008 ▶) and *Mercury* (Macrae *et al.*, 2006 ▶); software used to prepare material for publication: *SHELXTL*.

## Supplementary Material

Crystal structure: contains datablock(s) global, I. DOI: 10.1107/S1600536811021088/hg5047sup1.cif

Structure factors: contains datablock(s) I. DOI: 10.1107/S1600536811021088/hg5047Isup2.hkl

Additional supplementary materials:  crystallographic information; 3D view; checkCIF report

## Figures and Tables

**Table 1:** Hydrogen-bond geometry (Å, °)

*D*—H⋯*A*	*D*—H	H⋯*A*	*D*⋯*A*	*D*—H⋯*A*
N1—H1*A*⋯N3^i^	0.90	2.49	3.336 (6)	156
O3—H3⋯O4	0.86	1.94	2.748 (7)	155
O1*W*—H1*C*⋯O2*W*	0.82	1.79	2.611 (6)	173
O1*W*—H1*D*⋯N3^ii^	0.82	2.33	3.133 (6)	167
O2*W*—H2*A*⋯O4^iii^	0.82	2.10	2.889 (7)	160
O2*W*—H2*B*⋯O3*W*	0.82	1.97	2.774 (7)	168
O3*W*—H3*C*⋯O6^iv^	0.83	2.20	2.962 (7)	153
O3*W*—H3*D*⋯N5^v^	0.82	2.09	2.851 (6)	154

Symmetry codes: (i) 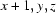; (ii) 
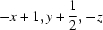
; (iii) 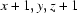; (iv) 
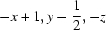
; (v) 
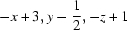
.

**Fig. 1.**
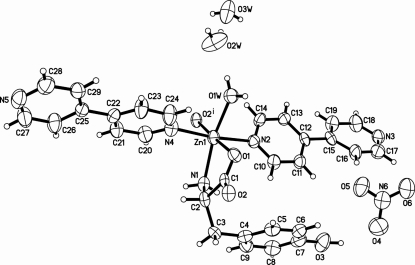
The asymmetric unit of the title compound. Displacement ellipsoids are drawn at the 50% probability level. [Symmetry code: (i) 2 - x, -1/2 + y, -z.]

**Fig. 2.**
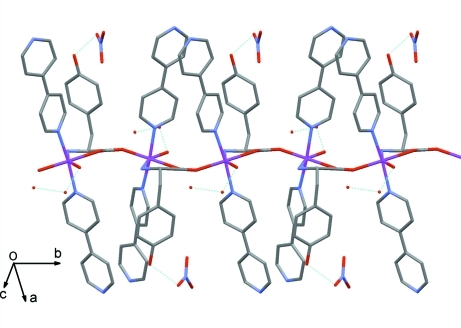
A view of the chain structure in the title compound. H atoms have been omitted for clarity. Dashed lines denote hydrogen bonds.

## supplementary crystallographic information

### Comment

There has been considerable interest in chiral coordination polymers, which exhibit potential applications in asymmetric catalysis and chiral separation (Kesanli & Lin, 2003). Self-assembly based on a mixed-ligand system containing both chiral and achiral ligands is an effective approach to the construction of chiral complexes (Dai *et al.*, 2005; Vaidhyanathan *et al.*, 2006; Zaworotko, 2001). Amino acids can be used as chiral building blocks, with their amino and carboxylate groups binding to metal ions in flexible modes (Lou *et al.*, 2005, 2007; Lou & Hong, 2008). We previously reported a chiral two-dimensional coordination polymer, [Cu_2_(L-tyr)_2_(4,4-bipy)(NO_3_)_2_(H_2_O)_2_]_n_, (II), which contains Cu^II^ ions, L-tyrosinate (L-tyr) and 4,4'-bipyridine (4,4-bipy) ligands in a 2:2:1 ratio (Zhang & Hu, 2009). Herein, we present the title compound, (I), a one-dimensional Zn(II) complex with a 1:1:2 ratio of the metal ion and organic ligands.

In (I), the Zn^II^ atom is six-coordinated by two O atoms and one N atom from two L-tyr ligands, two N atoms from two 4,4'-bipy ligands and one water molecule in a distorted octahedral geometry (Fig. 1). The L-tyr ligand bridges adjacent Zn atoms through the carboxylate group, forming a cationic chiral [Zn(L-tyr)(4,4-bipy)_2_(H_2_O)]_n_ chain extending along [0 1 0]. The separation between the Zn atoms in the chain is 5.441 (1) Å. The hydroxyl O atom of the phenol group is uncoordinated. The L-tyr ligand binds to the Zn atoms in a µ-(κ^3^*N*,*O*:*O*') mode, the same as that observed in (II). However, the 4,4'-bipy ligand adopts a monodentate terminal mode, different from the bridging mode in (II), which leads to a one-dimensional comb-like structure (Fig. 2) rather than a layer structure as shown in (II). Therefore, in the mixed-ligand system, the L-tyr ligand provides a chiral source, while the binding mode of the 4,4'-bipy ligand is an important factor affecting structural architectures. N—H···N, O—H···N and O—H···O hydrogen bonds (Table 1) link the cationic chains, nitrate anions and uncoordinated water molecules into a supramolecular network. Intrachain π–π interactions between the pyridine rings, *Cg*1···*Cg*2^i^ = 3.615 (4) Å, and interchain π–π interactions between the pyridine and benzene rings, *Cg*2···*Cg*3^ii^ = 3.636 (4) Å, stabilize the structure [*Cg*1, *Cg*2 and *Cg*3 are the centroids of the N3/C15–C19, N5/C25–C29 and C4–C9 rings. Symmetry codes: (i) 2 - *x*, -1/2 + *y*, -*z*; (ii) 1 + *x*, *y*, 1 + *z*].

### Experimental

Zn(NO_3_)_2_.6H_2_O (0.119 g, 0.4 mmol) and L-tyrosine (0.072 g, 0.4 mmol) were dissolved in hot water (25 ml) under stirring. To this solution 4,4'-bipyridine (0.062 g, 0.4 mmol) in methanol (10 ml) was added. The resulting solution was allowed to stand at room temperature and yellow crystals suitable for X-ray diffraction analysis were obtained after two weeks.

### Refinement

H atoms bonded to O atoms were located in a difference Fourier map and refined as riding atoms, with *U*_iso_(H) = 1.5*U*_eq_(O). Other H atoms were positioned geometrically and refined as riding atoms, with C—H = 0.93 (aromatic), 0.97 (CH_2_) and 0.98 (CH) Å and N—H = 0.90 Å and with *U*_iso_(H) = 1.2*U*_eq_(C, N).

### Crystal data

**Table:**

[Zn(C_9_H_10_NO_3_)(C_10_H_8_N_2_)_2_(H_2_O)]NO_3_·2H_2_O	*F*(000) = 700
*M**_r_* = 673.98	*D*_x_ = 1.487 Mg m^−^^3^
Monoclinic, *P*2_1_	Mo *K*α radiation, λ = 0.71073 Å
Hall symbol: P 2yb	Cell parameters from 5180 reflections
*a* = 12.737 (3) Å	θ = 2.0–25.1°
*b* = 10.351 (2) Å	µ = 0.88 mm^−^^1^
*c* = 12.921 (3) Å	*T* = 293 K
β = 117.897 (5)°	Column, yellow
*V* = 1505.5 (6) Å^3^	0.22 × 0.09 × 0.02 mm
*Z* = 2	

### Data collection

**Table:**

Bruker APEX CCD diffractometer	5199 independent reflections
Radiation source: sealed tube	3621 reflections with *I* > 2σ(*I*)
graphite	*R*_int_ = 0.048
φ and ω scans	θ_max_ = 25.1°, θ_min_ = 1.8°
Absorption correction: multi-scan (*SADABS*; Sheldrick, 1996)	*h* = −11→15
*T*_min_ = 0.830, *T*_max_ = 0.983	*k* = −12→12
7908 measured reflections	*l* = −15→8

### Refinement

**Table:**

Refinement on *F*^2^	Secondary atom site location: difference Fourier map
Least-squares matrix: full	Hydrogen site location: inferred from neighbouring sites
*R*[*F*^2^ > 2σ(*F*^2^)] = 0.056	H-atom parameters constrained
*wR*(*F*^2^) = 0.091	*w* = 1/[σ^2^(*F*_o_^2^) + (0.0098*P*)^2^] where *P* = (*F*_o_^2^ + 2*F*_c_^2^)/3
*S* = 0.97	(Δ/σ)_max_ < 0.001
5199 reflections	Δρ_max_ = 0.47 e Å^−^^3^
406 parameters	Δρ_min_ = −0.42 e Å^−^^3^
1 restraint	Absolute structure: Flack (1983), 2352 Friedel pairs
Primary atom site location: structure-invariant direct methods	Flack parameter: 0.045 (14)

### Fractional atomic coordinates and isotropic or equivalent isotropic displacement parameters (Å^2^)

**Table:**

	*x*	*y*	*z*	*U*_iso_*/*U*_eq_	
Zn1	0.98663 (5)	0.52948 (6)	0.05781 (6)	0.03552 (18)	
O1	0.9437 (3)	0.7189 (3)	−0.0165 (4)	0.0392 (10)	
O2	0.9765 (3)	0.8500 (3)	−0.1340 (4)	0.0357 (11)	
O3	0.4363 (3)	0.5483 (5)	−0.4711 (4)	0.0674 (14)	
H3	0.3776	0.6001	−0.4868	0.101*	
O4	0.2786 (5)	0.7124 (6)	−0.4484 (5)	0.0832 (19)	
O5	0.4065 (4)	0.7555 (4)	−0.2707 (4)	0.0655 (14)	
O6	0.2188 (4)	0.7763 (6)	−0.3267 (5)	0.0892 (19)	
O1W	0.9788 (3)	0.6216 (4)	0.2010 (4)	0.0521 (12)	
H1C	1.0047	0.6030	0.2708	0.078*	
H1D	0.9433	0.6905	0.1879	0.078*	
O2W	1.0732 (4)	0.5515 (5)	0.4204 (4)	0.0979 (18)	
H2A	1.1231	0.6008	0.4678	0.147*	
H2B	1.0447	0.5075	0.4538	0.147*	
O3W	0.9978 (4)	0.3726 (5)	0.5315 (4)	0.0868 (18)	
H3C	0.9287	0.3486	0.4905	0.130*	
H3D	1.0422	0.3110	0.5413	0.130*	
N1	0.9777 (3)	0.5024 (4)	−0.1113 (4)	0.0343 (13)	
H1A	1.0334	0.4451	−0.1055	0.041*	
H1B	0.9060	0.4704	−0.1617	0.041*	
N2	0.7974 (4)	0.4738 (4)	−0.0048 (5)	0.0357 (13)	
N3	0.1952 (4)	0.3563 (6)	−0.1416 (5)	0.0517 (15)	
N4	1.1758 (4)	0.5685 (4)	0.1397 (5)	0.0419 (15)	
N5	1.7923 (4)	0.7197 (6)	0.3878 (5)	0.0560 (17)	
N6	0.3008 (6)	0.7476 (6)	−0.3472 (6)	0.0569 (18)	
C1	0.9696 (4)	0.7405 (5)	−0.0963 (5)	0.0303 (14)	
C2	0.9972 (4)	0.6270 (5)	−0.1564 (5)	0.0323 (15)	
H2	1.0823	0.6317	−0.1328	0.039*	
C3	0.9319 (4)	0.6385 (6)	−0.2899 (5)	0.0385 (15)	
H3A	0.9466	0.7234	−0.3121	0.046*	
H3B	0.9636	0.5750	−0.3230	0.046*	
C4	0.7991 (5)	0.6185 (6)	−0.3409 (5)	0.0398 (16)	
C5	0.7253 (5)	0.7113 (6)	−0.3317 (5)	0.0419 (16)	
H5	0.7578	0.7901	−0.2967	0.050*	
C6	0.6048 (5)	0.6907 (6)	−0.3729 (5)	0.0453 (17)	
H6	0.5583	0.7541	−0.3633	0.054*	
C7	0.5535 (6)	0.5759 (6)	−0.4284 (6)	0.0501 (19)	
C8	0.6243 (6)	0.4824 (6)	−0.4417 (6)	0.0502 (19)	
H8	0.5902	0.4062	−0.4814	0.060*	
C9	0.7457 (5)	0.5019 (6)	−0.3962 (5)	0.0447 (18)	
H9	0.7926	0.4366	−0.4024	0.054*	
C10	0.7067 (4)	0.5145 (7)	−0.1040 (5)	0.0410 (15)	
H10	0.7238	0.5597	−0.1564	0.049*	
C11	0.5883 (5)	0.4938 (5)	−0.1342 (5)	0.0410 (18)	
H11	0.5285	0.5253	−0.2046	0.049*	
C12	0.5601 (5)	0.4255 (5)	−0.0580 (5)	0.0331 (15)	
C13	0.6544 (5)	0.3816 (5)	0.0445 (5)	0.0402 (16)	
H13	0.6397	0.3333	0.0970	0.048*	
C14	0.7698 (5)	0.4086 (5)	0.0695 (6)	0.0426 (17)	
H14	0.8311	0.3807	0.1406	0.051*	
C15	0.4342 (5)	0.4008 (5)	−0.0871 (5)	0.0371 (16)	
C16	0.3420 (5)	0.4794 (6)	−0.1647 (6)	0.051 (2)	
H16	0.3583	0.5509	−0.1985	0.062*	
C17	0.2266 (6)	0.4506 (7)	−0.1911 (6)	0.061 (2)	
H17	0.1665	0.5008	−0.2475	0.073*	
C18	0.2840 (5)	0.2878 (6)	−0.0652 (6)	0.055 (2)	
H18	0.2659	0.2222	−0.0269	0.066*	
C19	0.4017 (5)	0.3041 (5)	−0.0364 (6)	0.0474 (18)	
H19	0.4589	0.2492	0.0175	0.057*	
C20	1.2537 (5)	0.4846 (6)	0.1388 (6)	0.0489 (19)	
H20	1.2252	0.4049	0.1035	0.059*	
C21	1.3747 (5)	0.5066 (6)	0.1865 (5)	0.0451 (18)	
H21	1.4246	0.4441	0.1815	0.054*	
C22	1.4206 (5)	0.6245 (6)	0.2425 (5)	0.0365 (15)	
C23	1.3409 (5)	0.7119 (6)	0.2452 (6)	0.0525 (19)	
H23	1.3669	0.7916	0.2816	0.063*	
C24	1.2206 (5)	0.6801 (6)	0.1929 (6)	0.0532 (19)	
H24	1.1682	0.7410	0.1955	0.064*	
C25	1.5496 (5)	0.6566 (5)	0.2940 (5)	0.0361 (15)	
C26	1.6280 (5)	0.5827 (6)	0.2715 (6)	0.054 (2)	
H26	1.6011	0.5100	0.2240	0.065*	
C27	1.7453 (6)	0.6179 (7)	0.3199 (7)	0.063 (2)	
H27	1.7956	0.5663	0.3036	0.076*	
C28	1.7173 (6)	0.7898 (7)	0.4097 (6)	0.056 (2)	
H28	1.7472	0.8616	0.4580	0.068*	
C29	1.5978 (5)	0.7626 (6)	0.3650 (5)	0.0449 (17)	
H29	1.5498	0.8160	0.3829	0.054*	

### Atomic displacement parameters (Å^2^)

**Table:**

	*U*^11^	*U*^22^	*U*^33^	*U*^12^	*U*^13^	*U*^23^
Zn1	0.0329 (3)	0.0303 (3)	0.0443 (4)	0.0015 (4)	0.0188 (3)	0.0008 (5)
O1	0.042 (2)	0.037 (2)	0.044 (3)	0.0051 (18)	0.025 (2)	−0.001 (2)
O2	0.032 (2)	0.027 (2)	0.049 (3)	−0.0040 (18)	0.019 (2)	0.000 (2)
O3	0.036 (2)	0.078 (4)	0.073 (3)	−0.007 (3)	0.013 (2)	0.006 (3)
O4	0.071 (4)	0.107 (5)	0.063 (4)	−0.003 (3)	0.024 (3)	−0.014 (4)
O5	0.041 (3)	0.070 (3)	0.070 (4)	−0.004 (2)	0.012 (3)	−0.005 (3)
O6	0.049 (3)	0.129 (5)	0.085 (5)	0.014 (3)	0.027 (3)	−0.001 (4)
O1W	0.055 (3)	0.056 (3)	0.045 (3)	0.019 (2)	0.023 (2)	0.004 (2)
O2W	0.099 (3)	0.102 (5)	0.057 (4)	−0.033 (4)	0.007 (3)	0.006 (4)
O3W	0.048 (3)	0.079 (3)	0.113 (5)	0.006 (3)	0.021 (3)	0.002 (4)
N1	0.029 (2)	0.026 (3)	0.045 (3)	−0.001 (2)	0.014 (2)	−0.004 (2)
N2	0.037 (3)	0.033 (3)	0.039 (4)	0.004 (2)	0.019 (3)	0.004 (2)
N3	0.033 (3)	0.073 (4)	0.051 (4)	−0.001 (3)	0.020 (3)	−0.004 (3)
N4	0.038 (3)	0.028 (3)	0.057 (4)	0.000 (2)	0.020 (3)	0.004 (2)
N5	0.037 (3)	0.064 (4)	0.061 (5)	−0.006 (3)	0.018 (3)	0.004 (4)
N6	0.053 (4)	0.050 (4)	0.067 (6)	−0.005 (3)	0.027 (4)	0.000 (4)
C1	0.018 (3)	0.034 (4)	0.039 (4)	0.002 (2)	0.013 (3)	−0.002 (3)
C2	0.028 (3)	0.025 (3)	0.049 (4)	0.003 (3)	0.022 (3)	0.002 (3)
C3	0.037 (4)	0.049 (4)	0.038 (4)	0.002 (3)	0.024 (3)	0.003 (3)
C4	0.045 (4)	0.041 (4)	0.030 (4)	−0.002 (3)	0.015 (3)	0.003 (3)
C5	0.045 (4)	0.039 (4)	0.039 (4)	−0.008 (3)	0.017 (3)	−0.004 (3)
C6	0.047 (4)	0.049 (4)	0.037 (4)	0.007 (3)	0.017 (3)	0.008 (3)
C7	0.043 (4)	0.065 (5)	0.037 (4)	−0.006 (4)	0.014 (3)	0.008 (3)
C8	0.056 (5)	0.044 (4)	0.049 (5)	−0.013 (3)	0.023 (4)	−0.009 (3)
C9	0.051 (4)	0.042 (5)	0.046 (4)	0.006 (3)	0.027 (3)	0.001 (3)
C10	0.038 (3)	0.039 (4)	0.053 (4)	0.001 (4)	0.027 (3)	0.016 (4)
C11	0.032 (3)	0.048 (5)	0.044 (4)	0.002 (3)	0.018 (3)	0.006 (3)
C12	0.032 (3)	0.027 (3)	0.047 (5)	0.000 (3)	0.023 (3)	−0.008 (3)
C13	0.040 (4)	0.042 (4)	0.040 (4)	0.006 (3)	0.020 (3)	0.008 (3)
C14	0.037 (4)	0.044 (4)	0.040 (5)	0.011 (3)	0.012 (3)	0.007 (3)
C15	0.033 (4)	0.038 (4)	0.051 (5)	−0.005 (3)	0.028 (3)	−0.008 (3)
C16	0.033 (4)	0.067 (5)	0.061 (5)	0.003 (3)	0.028 (3)	0.014 (4)
C17	0.054 (5)	0.079 (5)	0.060 (6)	0.011 (4)	0.036 (4)	0.022 (4)
C18	0.044 (4)	0.049 (4)	0.079 (6)	−0.009 (4)	0.035 (4)	0.002 (4)
C19	0.038 (4)	0.040 (4)	0.062 (5)	−0.010 (3)	0.022 (4)	0.001 (3)
C20	0.043 (4)	0.045 (4)	0.058 (5)	−0.008 (3)	0.022 (4)	−0.014 (3)
C21	0.038 (3)	0.042 (5)	0.057 (4)	−0.001 (3)	0.023 (3)	−0.003 (4)
C22	0.033 (4)	0.036 (3)	0.036 (4)	0.001 (3)	0.013 (3)	0.004 (3)
C23	0.034 (4)	0.036 (4)	0.079 (6)	−0.004 (3)	0.018 (4)	−0.014 (4)
C24	0.042 (4)	0.034 (4)	0.078 (6)	0.005 (3)	0.023 (4)	−0.005 (4)
C25	0.033 (3)	0.040 (4)	0.036 (4)	−0.001 (3)	0.016 (3)	0.009 (3)
C26	0.037 (4)	0.053 (5)	0.071 (6)	−0.010 (3)	0.025 (4)	−0.007 (4)
C27	0.043 (4)	0.073 (5)	0.078 (6)	0.002 (4)	0.033 (4)	−0.008 (5)
C28	0.049 (5)	0.062 (5)	0.053 (6)	−0.005 (4)	0.020 (5)	0.004 (4)
C29	0.039 (4)	0.052 (4)	0.045 (5)	−0.005 (3)	0.021 (3)	−0.003 (4)

### Geometric parameters (Å, °)

**Table:**

Zn1—O1	2.139 (4)	C6—C7	1.383 (7)
Zn1—O2^i^	2.052 (4)	C6—H6	0.9300
Zn1—O1W	2.125 (4)	C7—C8	1.389 (8)
Zn1—N1	2.153 (4)	C8—C9	1.388 (7)
Zn1—N2	2.229 (4)	C8—H8	0.9300
Zn1—N4	2.169 (5)	C9—H9	0.9300
O1—C1	1.240 (6)	C10—C11	1.386 (6)
O2—C1	1.252 (6)	C10—H10	0.9300
O3—C7	1.357 (6)	C11—C12	1.388 (7)
O3—H3	0.8600	C11—H11	0.9300
O4—N6	1.256 (7)	C12—C13	1.383 (7)
O5—N6	1.245 (6)	C12—C15	1.488 (7)
O6—N6	1.229 (6)	C13—C14	1.377 (7)
O1W—H1C	0.8248	C13—H13	0.9300
O1W—H1D	0.8190	C14—H14	0.9300
O2W—H2A	0.8222	C15—C19	1.363 (8)
O2W—H2B	0.8196	C15—C16	1.395 (8)
O3W—H3C	0.8253	C16—C17	1.377 (8)
O3W—H3D	0.8211	C16—H16	0.9300
N1—C2	1.483 (6)	C17—H17	0.9300
N1—H1A	0.9000	C18—C19	1.376 (7)
N1—H1B	0.9000	C18—H18	0.9300
N2—C10	1.330 (6)	C19—H19	0.9300
N2—C14	1.349 (7)	C20—C21	1.384 (7)
N3—C18	1.309 (7)	C20—H20	0.9300
N3—C17	1.327 (7)	C21—C22	1.399 (8)
N4—C20	1.323 (6)	C21—H21	0.9300
N4—C24	1.328 (6)	C22—C23	1.373 (7)
N5—C27	1.321 (8)	C22—C25	1.494 (7)
N5—C28	1.331 (8)	C23—C24	1.395 (7)
C1—C2	1.537 (7)	C23—H23	0.9300
C2—C3	1.529 (7)	C24—H24	0.9300
C2—H2	0.9800	C25—C29	1.377 (7)
C3—C4	1.514 (7)	C25—C26	1.392 (7)
C3—H3A	0.9700	C26—C27	1.372 (8)
C3—H3B	0.9700	C26—H26	0.9300
C4—C5	1.388 (7)	C27—H27	0.9300
C4—C9	1.405 (8)	C28—C29	1.381 (7)
C5—C6	1.386 (7)	C28—H28	0.9300
C5—H5	0.9300	C29—H29	0.9300
			
O2^i^—Zn1—O1W	94.70 (16)	C9—C8—C7	120.4 (6)
O2^i^—Zn1—O1	177.13 (17)	C9—C8—H8	119.8
O1W—Zn1—O1	82.75 (16)	C7—C8—H8	119.8
O2^i^—Zn1—N1	105.10 (16)	C8—C9—C4	121.2 (6)
O1W—Zn1—N1	160.17 (15)	C8—C9—H9	119.4
O1—Zn1—N1	77.43 (16)	C4—C9—H9	119.4
O2^i^—Zn1—N4	88.95 (15)	N2—C10—C11	124.3 (5)
O1W—Zn1—N4	89.17 (18)	N2—C10—H10	117.9
O1—Zn1—N4	92.32 (15)	C11—C10—H10	117.9
N1—Zn1—N4	92.21 (18)	C10—C11—C12	119.1 (6)
O2^i^—Zn1—N2	84.61 (15)	C10—C11—H11	120.5
O1W—Zn1—N2	87.08 (16)	C12—C11—H11	120.5
O1—Zn1—N2	93.93 (15)	C13—C12—C11	116.7 (5)
N1—Zn1—N2	93.61 (17)	C13—C12—C15	122.4 (6)
N4—Zn1—N2	172.26 (19)	C11—C12—C15	120.9 (5)
C1—O1—Zn1	115.3 (4)	C14—C13—C12	120.7 (6)
C1—O2—Zn1^ii^	132.4 (4)	C14—C13—H13	119.6
C7—O3—H3	129.2	C12—C13—H13	119.6
Zn1—O1W—H1C	133.6	N2—C14—C13	122.6 (6)
Zn1—O1W—H1D	117.2	N2—C14—H14	118.7
H1C—O1W—H1D	109.1	C13—C14—H14	118.7
H2A—O2W—H2B	109.4	C19—C15—C16	115.5 (5)
H3C—O3W—H3D	108.4	C19—C15—C12	122.6 (6)
C2—N1—Zn1	110.2 (3)	C16—C15—C12	121.8 (5)
C2—N1—H1A	109.6	C17—C16—C15	119.6 (6)
Zn1—N1—H1A	109.6	C17—C16—H16	120.2
C2—N1—H1B	109.6	C15—C16—H16	120.2
Zn1—N1—H1B	109.6	N3—C17—C16	124.6 (6)
H1A—N1—H1B	108.1	N3—C17—H17	117.7
C10—N2—C14	116.5 (5)	C16—C17—H17	117.7
C10—N2—Zn1	124.9 (4)	N3—C18—C19	125.7 (6)
C14—N2—Zn1	118.0 (4)	N3—C18—H18	117.1
C18—N3—C17	114.5 (6)	C19—C18—H18	117.1
C20—N4—C24	115.7 (5)	C15—C19—C18	119.9 (6)
C20—N4—Zn1	122.7 (4)	C15—C19—H19	120.0
C24—N4—Zn1	121.6 (4)	C18—C19—H19	120.0
C27—N5—C28	115.4 (6)	N4—C20—C21	124.8 (6)
O6—N6—O5	121.6 (7)	N4—C20—H20	117.6
O6—N6—O4	119.7 (7)	C21—C20—H20	117.6
O5—N6—O4	118.7 (7)	C20—C21—C22	118.9 (6)
O1—C1—O2	125.5 (5)	C20—C21—H21	120.6
O1—C1—C2	119.6 (5)	C22—C21—H21	120.6
O2—C1—C2	114.9 (5)	C23—C22—C21	116.9 (5)
N1—C2—C3	114.0 (5)	C23—C22—C25	121.0 (5)
N1—C2—C1	110.3 (4)	C21—C22—C25	122.1 (5)
C3—C2—C1	112.2 (5)	C22—C23—C24	119.4 (6)
N1—C2—H2	106.6	C22—C23—H23	120.3
C3—C2—H2	106.6	C24—C23—H23	120.3
C1—C2—H2	106.6	N4—C24—C23	124.3 (6)
C4—C3—C2	112.9 (4)	N4—C24—H24	117.8
C4—C3—H3A	109.0	C23—C24—H24	117.8
C2—C3—H3A	109.0	C29—C25—C26	115.9 (5)
C4—C3—H3B	109.0	C29—C25—C22	121.9 (5)
C2—C3—H3B	109.0	C26—C25—C22	122.1 (6)
H3A—C3—H3B	107.8	C27—C26—C25	119.6 (6)
C5—C4—C9	116.9 (5)	C27—C26—H26	120.2
C5—C4—C3	122.1 (5)	C25—C26—H26	120.2
C9—C4—C3	121.0 (5)	N5—C27—C26	124.9 (6)
C6—C5—C4	122.2 (6)	N5—C27—H27	117.5
C6—C5—H5	118.9	C26—C27—H27	117.5
C4—C5—H5	118.9	N5—C28—C29	124.1 (7)
C7—C6—C5	120.1 (6)	N5—C28—H28	118.0
C7—C6—H6	120.0	C29—C28—H28	118.0
C5—C6—H6	120.0	C25—C29—C28	120.1 (6)
O3—C7—C6	123.7 (6)	C25—C29—H29	120.0
O3—C7—C8	117.2 (6)	C28—C29—H29	120.0
C6—C7—C8	119.1 (6)		
			
O1W—Zn1—O1—C1	158.7 (4)	C5—C4—C9—C8	1.5 (9)
N1—Zn1—O1—C1	−21.9 (4)	C3—C4—C9—C8	179.1 (6)
N4—Zn1—O1—C1	69.8 (4)	C14—N2—C10—C11	−0.1 (9)
N2—Zn1—O1—C1	−114.8 (4)	Zn1—N2—C10—C11	−170.7 (5)
O2^i^—Zn1—N1—C2	−158.5 (3)	N2—C10—C11—C12	−0.6 (10)
O1W—Zn1—N1—C2	24.7 (6)	C10—C11—C12—C13	−0.3 (8)
O1—Zn1—N1—C2	22.9 (3)	C10—C11—C12—C15	−179.9 (5)
N4—Zn1—N1—C2	−69.0 (3)	C11—C12—C13—C14	1.8 (8)
N2—Zn1—N1—C2	116.1 (3)	C15—C12—C13—C14	−178.7 (5)
O2^i^—Zn1—N2—C10	−149.5 (5)	C10—N2—C14—C13	1.7 (8)
O1W—Zn1—N2—C10	115.5 (5)	Zn1—N2—C14—C13	172.9 (4)
O1—Zn1—N2—C10	32.9 (5)	C12—C13—C14—N2	−2.6 (9)
N1—Zn1—N2—C10	−44.7 (5)	C13—C12—C15—C19	−20.4 (8)
O2^i^—Zn1—N2—C14	40.0 (4)	C11—C12—C15—C19	159.2 (6)
O1W—Zn1—N2—C14	−55.0 (4)	C13—C12—C15—C16	156.8 (6)
O1—Zn1—N2—C14	−137.5 (4)	C11—C12—C15—C16	−23.7 (8)
N1—Zn1—N2—C14	144.9 (4)	C19—C15—C16—C17	−4.3 (9)
O2^i^—Zn1—N4—C20	46.0 (5)	C12—C15—C16—C17	178.3 (6)
O1W—Zn1—N4—C20	140.7 (5)	C18—N3—C17—C16	−1.6 (10)
O1—Zn1—N4—C20	−136.6 (5)	C15—C16—C17—N3	4.7 (11)
N1—Zn1—N4—C20	−59.1 (5)	C17—N3—C18—C19	−1.8 (10)
O2^i^—Zn1—N4—C24	−135.0 (5)	C16—C15—C19—C18	1.4 (9)
O1W—Zn1—N4—C24	−40.3 (5)	C12—C15—C19—C18	178.7 (5)
O1—Zn1—N4—C24	42.4 (5)	N3—C18—C19—C15	1.8 (10)
N1—Zn1—N4—C24	119.9 (5)	C24—N4—C20—C21	−1.2 (10)
Zn1—O1—C1—O2	−163.6 (4)	Zn1—N4—C20—C21	177.9 (5)
Zn1—O1—C1—C2	15.9 (6)	N4—C20—C21—C22	1.4 (10)
Zn1^ii^—O2—C1—O1	26.8 (8)	C20—C21—C22—C23	−0.6 (9)
Zn1^ii^—O2—C1—C2	−152.8 (4)	C20—C21—C22—C25	−178.7 (6)
Zn1—N1—C2—C3	−149.2 (3)	C21—C22—C23—C24	−0.2 (10)
Zn1—N1—C2—C1	−21.9 (5)	C25—C22—C23—C24	178.0 (6)
O1—C1—C2—N1	4.6 (7)	C20—N4—C24—C23	0.3 (10)
O2—C1—C2—N1	−175.8 (4)	Zn1—N4—C24—C23	−178.8 (5)
O1—C1—C2—C3	133.0 (5)	C22—C23—C24—N4	0.4 (11)
O2—C1—C2—C3	−47.4 (6)	C23—C22—C25—C29	12.6 (9)
N1—C2—C3—C4	56.5 (6)	C21—C22—C25—C29	−169.3 (6)
C1—C2—C3—C4	−69.8 (6)	C23—C22—C25—C26	−166.2 (6)
C2—C3—C4—C5	73.9 (7)	C21—C22—C25—C26	11.9 (9)
C2—C3—C4—C9	−103.5 (6)	C29—C25—C26—C27	0.2 (9)
C9—C4—C5—C6	1.0 (9)	C22—C25—C26—C27	179.0 (6)
C3—C4—C5—C6	−176.5 (5)	C28—N5—C27—C26	0.7 (11)
C4—C5—C6—C7	−1.8 (9)	C25—C26—C27—N5	−0.4 (11)
C5—C6—C7—O3	180.0 (5)	C27—N5—C28—C29	−0.9 (11)
C5—C6—C7—C8	0.1 (9)	C26—C25—C29—C28	−0.3 (9)
O3—C7—C8—C9	−177.5 (5)	C22—C25—C29—C28	−179.2 (6)
C6—C7—C8—C9	2.4 (10)	N5—C28—C29—C25	0.7 (11)
C7—C8—C9—C4	−3.2 (9)		

Symmetry codes: (i) −*x*+2, *y*−1/2, −*z*; (ii) −*x*+2, *y*+1/2, −*z*.

### Hydrogen-bond geometry (Å, °)

**Table:**

*D*—H···*A*	*D*—H	H···*A*	*D*···*A*	*D*—H···*A*
N1—H1A···N3^iii^	0.90	2.49	3.336 (6)	156
O3—H3···O4	0.86	1.94	2.748 (7)	155
O1W—H1C···O2W	0.82	1.79	2.611 (6)	173
O1W—H1D···N3^iv^	0.82	2.33	3.133 (6)	167
O2W—H2A···O4^v^	0.82	2.10	2.889 (7)	160
O2W—H2B···O3W	0.82	1.97	2.774 (7)	168
O3W—H3C···O6^vi^	0.83	2.20	2.962 (7)	153
O3W—H3D···N5^vii^	0.82	2.09	2.851 (6)	154

Symmetry codes: (iii) *x*+1, *y*, *z*; (iv) −*x*+1, *y*+1/2, −*z*; (v) *x*+1, *y*, *z*+1; (vi) −*x*+1, *y*−1/2, −*z*; (vii) −*x*+3, *y*−1/2, −*z*+1.
